# Editorial: Community Series: Avian Models for Social Cohesion, Volume II

**DOI:** 10.3389/fphys.2023.1215766

**Published:** 2023-05-12

**Authors:** Gergely Zachar, András Csillag

**Affiliations:** Department of Anatomy, Histology and Embryology, Semmelweis University, Budapest, Hungary

**Keywords:** birds, social, brain, mesolimbic, imprinting

The study of the physiological basis of social behavior is a fascinating field that has long intrigued researchers. Avian models have played a significant role in advancing our understanding of social cohesion, and the Research Topic of “Community Series: Avian Models for Social Cohesion, Volume II.” published by Frontiers in Physiology is a useful resource for anyone interested in this field of research.

Birds have proven to be useful tools for studying social cohesion because they exhibit complex social behaviors, including communication, social binding, and social learning comparable to, sometimes even exceeding, those of mammals. The studies compiled in this Research Topic provide valuable insights into how avian models can help us understand the mechanisms underlying social cohesion.

The four studies comprising the present Research Topic focus on diverse models at different levels of mechanisms and explanation. Two of the studies tackle the question of imprinting, an arguably specific form of social learning that leads to social cohesion between the parent and the offspring. They exploit the fact that imprinting is the result of a complex interaction between genetically influenced preferences, innate yet developmentally modified predispositions, and an early learning process. This well-established and widely studied model behavior is ideal to distinguish the role of different elements in the formation of social bonds.

The first study investigates such an interaction between *in ovo* developmental effects and the postnatal learning procedure. Serizawa et al. show that inhibiting thyroid hormone biosynthesis during late embryonic development leads to a deteriorated imprinting. There is a narrow time window in precocious birds after hatching, for the formation of the social bond between the parent and the offspring. The authors argue that delayed cognitive development, or impaired preference (probably even habituation) toward the imprinting stimulus due to reduced embryonic thyroid hormone levels might interfere with the post-hatch formation of social bond. The dissociation of acquired and innate (genetic and developmental) components of a complex behavior such as predisposed learning is notoriously difficult, if not impossible, in mammals. Such a direct link between embryonic development and early learning makes the chick a prospective model for studying the interaction between an innate physiological trait and the learning process based on it.

Cherepov et al. also study the interaction of the innate and learned components of imprinting. Newly hatched chicks show unconditional preferences toward natural, as compared to artificial, stimuli during imprinting. The second study focuses on the behavioral and neural levels of such interaction. The authors show that chicks can indeed learn to follow artificial stimuli in the short term. Moreover, an artificial stimulus elicits higher immediate early gene activation in some visual (hyperpallium apicale), associative (medial mesopallium) and memory-related (hippocampus, arcopallium, intermediate medial mesopallium) brain regions. Furthermore, the artificial stimulus results in a stronger preference toward the imprinted stimulus in the short term, however in the longer term (24 h) chicks exhibit weaker memory or even habituation to the stimulus. Such a habituation is not unlike the one that the chicks show in the study of Serizawa et al. Once again, the study by Cherepov et al. indicates that a healthy innate preference toward social stimuli is essential for the formation of social bonds later on.

The third study also pursues the understanding of the neural foundation of social and cognitive behaviors in birds. Fujita et al. describes the functional connection between the midbrain dopaminergic system and the serotonergic neurons of the raphe. The mesolimbic dopaminergic system has a close relationship with the so-called social brain network (Newman, 1999), and together they form a phylogenetically conservative network responsible for most of decision making in social context (O’Connell and Hofmann, 2011). Nevertheless, such a network is not independent from the rest of the brain, and the rest of the neurotransmitter systems. By demonstrating that the dopaminergic nuclei of an avian species (the domestic chicken) contain neurons that express 5-HTR1A and 5-HTR1B receptors, the authors made an important step toward generalization of the notion of serotoninergic input to the mesolimbic dopaminergic system, possibly also modulating social behavior. It is evident from the paper that the dopaminergic neurons do not express the serotonin receptors, therefore, the precise mechanism of the interaction between the two neurotransmitter systems needs to be further investigated.

Last but not least, the study of Polzin et al. reports that stimulation of the mu opioid receptors in the medial preoptic area or nucleus accumbens increased both singing behavior and the birds’ sensitivity to a rewarding stimulus, in European starlings. These regions are among the main targets of the midbrain dopaminergic nuclei. The findings of the study suggest that mu opioid receptor signaling in the medial preoptic area and nucleus accumbens plays a crucial role in promoting vocal communication and in regulating prosocial behavior and reward processing in birds. The study provides valuable insights into the neural mechanisms connecting the well-studied singing behavior with the less extensively studied elements of the social decision-making network.

Overall, the Research Topic “Community series: avian models for social cohesion, volume II” provides a valuable contribution to the field of social behavior research, and sheds light on the phylogenetically conservative social motivation systems in the avian brain. The studies included in this Research Topic demonstrate the importance of avian models for understanding social cohesion, and the potential of avian models in general. As we continue to explore the complex world of social bonds, avian models will undoubtedly play an increasingly important role in advancing our understanding of social cohesion and its disorders.
